# Supplementary material for: Lack of evidence for participation of TMEM150C in sensory mechanotransduction
Source: J Gen Physiol. 2022 Oct 18;154(12):e202213098. doi: 10.1085/jgp.202213098 (PMC9582506; doi:10.1085/jgp.202213098)
Supplement: SourceData F2 — is the source file for Fig. 2. [file JGP_202213098_SourceDataF2.pdf]

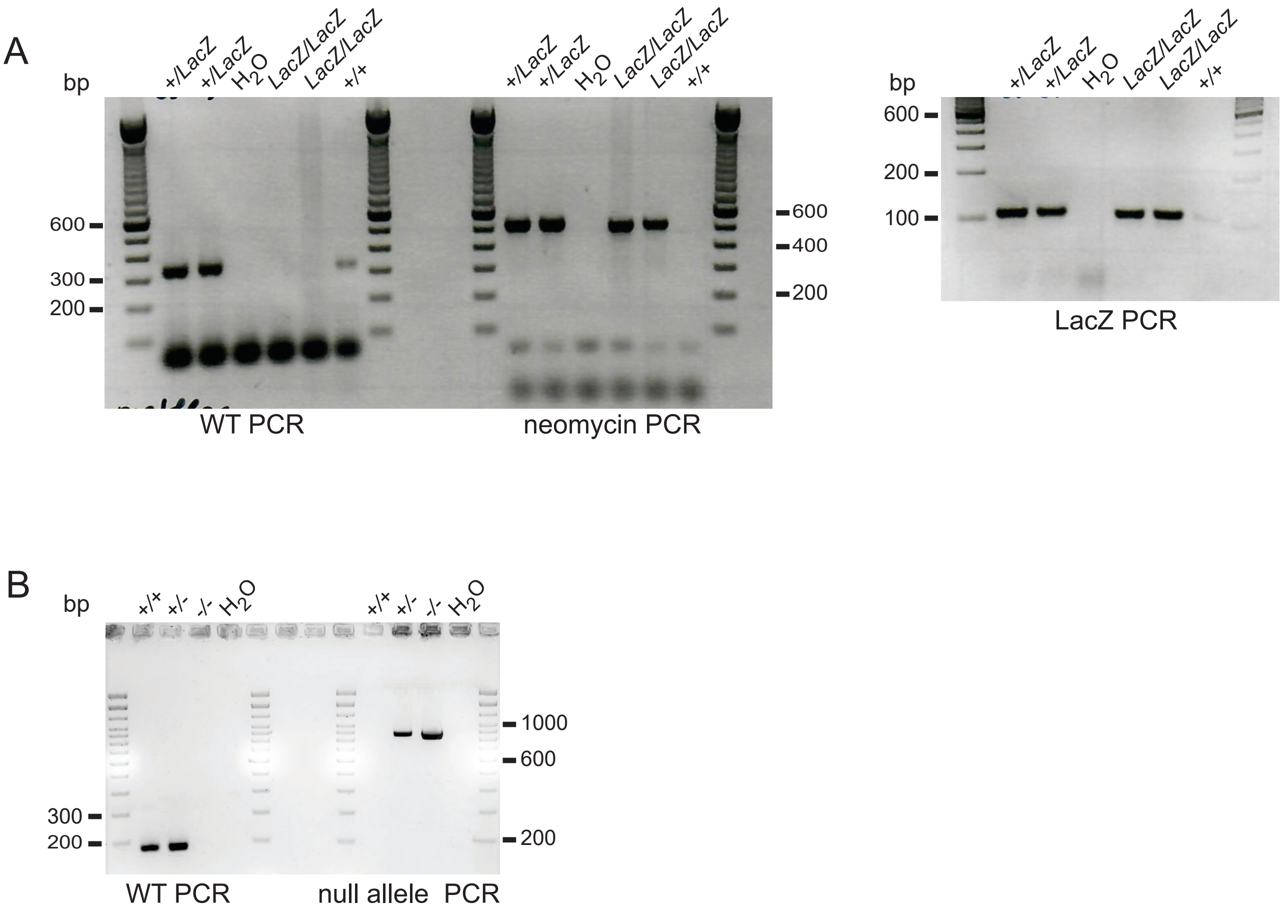

SourceDataFig2: Genotyping gels performed on gDNA from *Tmem150c*^{LacZ} KOMP and *Tmem150c* KO mice shown in Fig 2A and E.

(A) Uncropped pictures of the genotyping gels shown in the manuscript in Fig 2A: amplification of fragments for the (A, left) wild-type allele (336 bp), (A, middle) neomycin cassette (550 bp) and (A, right) LacZ cassette (108 bp) from gDNA obtained fromTmem150c KOMP mice: *Tmem150c*$^{+/+}$, *Tmem150c*$^{+/LacZ}$ and *Tmem150c*$^{LacZ/LacZ}$ (*LacZ/LacZ*). **(B)** Uncropped pictures of the genotyping gels shown in the manuscript in Fig 2E: amplification of fragments for the (A, left) wild-type allele (179 bp) and (A, right) null allele (886 bp) from gDNA obtained from*Tmem150c*$^{+/+}$, *Tmem150c*$^{+/-}$ and *Tmem150c*$^{-/-}$ mice. H_2O: water control for the RT-PCR. bp: base pair

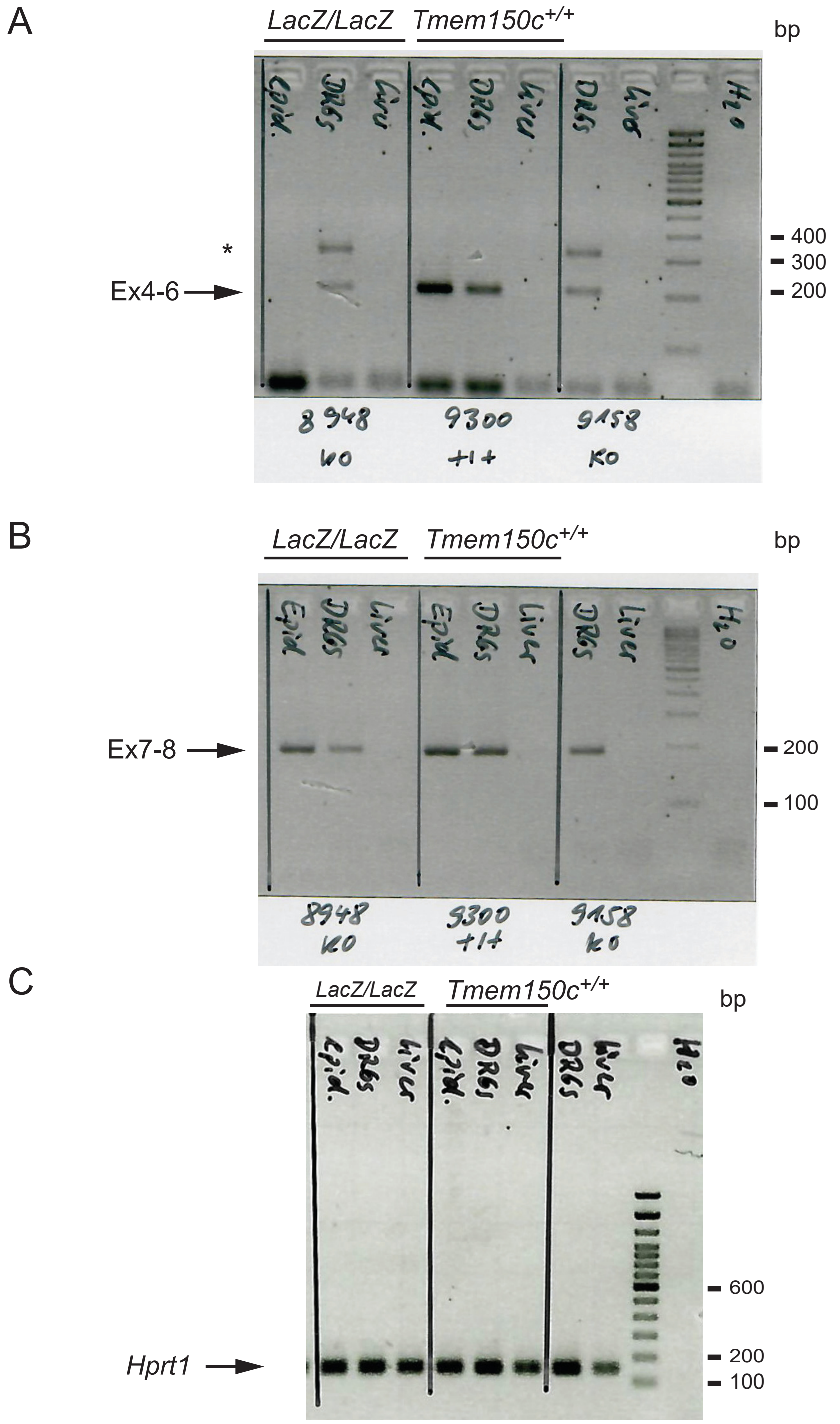

SourceDataFig 2: RT-PCR gels performed on cDNA from *Tmem150c^{LacZ}* KOMP mice of Panel C. **(A-C)** Uncropped pictures of the RT-PCR gels shown in the manuscript in Fig 2C: Amplification of part of Exons 4-6 (A) and Exons 7-8 (B) of *Tmem150c* cDNA in various tissues of *Tmem150c$^{+/+}$* and *Tmem150-c$^{LacZ/LacZ}$* (*LacZ/LacZ*) mice (epid: epididymis; DRGS: dorsal root ganglia). **(C)** The house keeping gene *Hprt1* was used as control. H_2O: water control. * unexpected band.

A

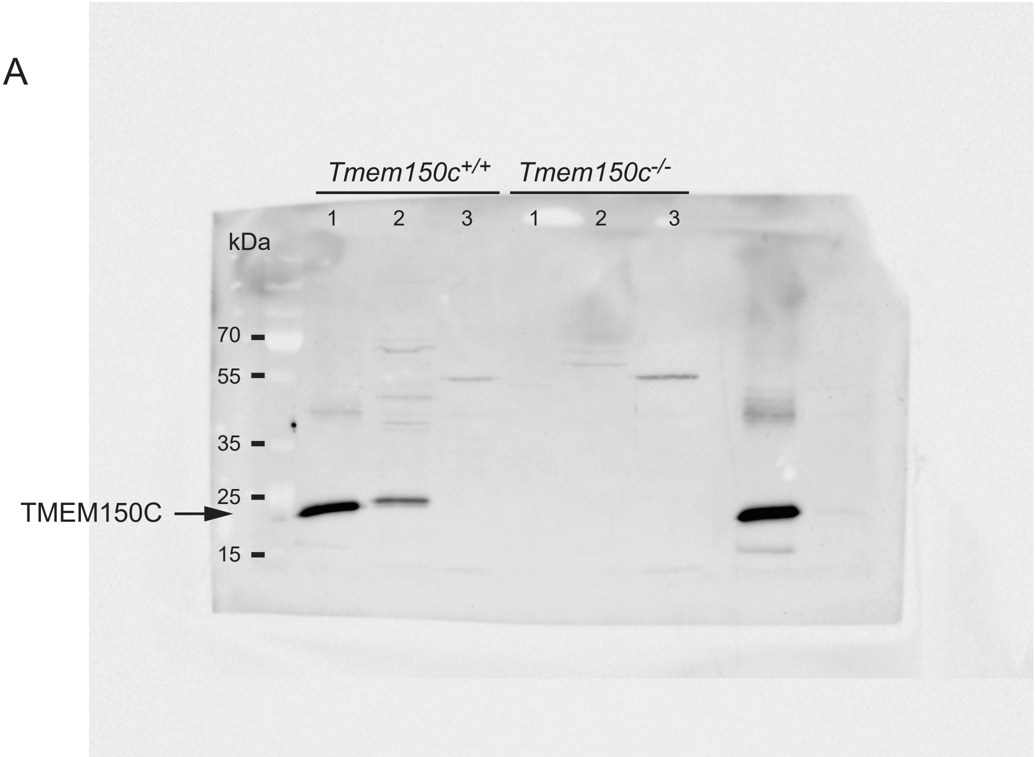

B

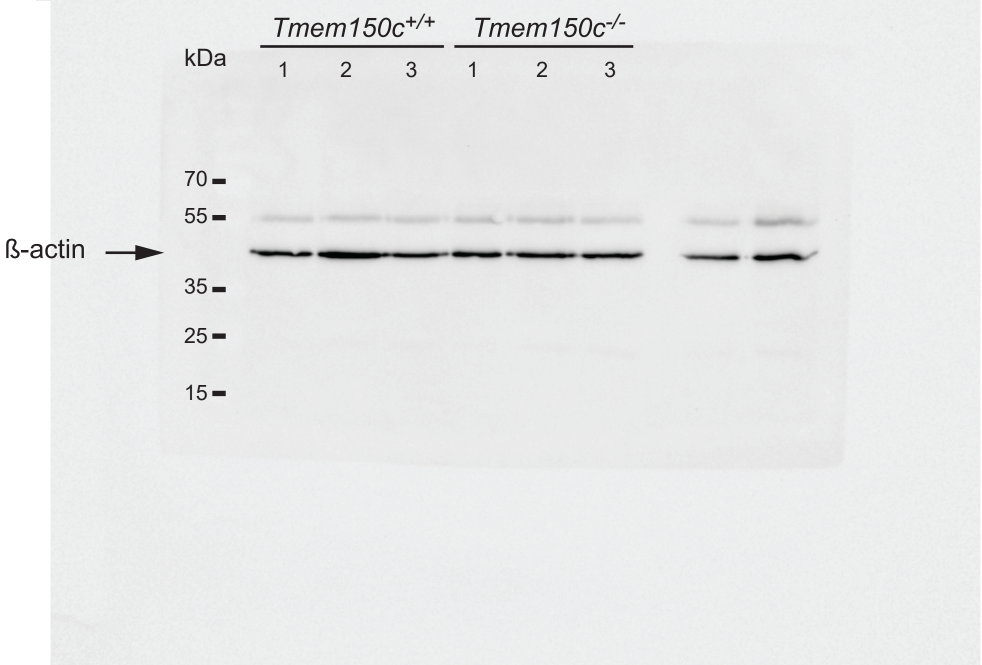

SourceDataFig 2: Uncropped Western blots from Panel F.

(A-B) Uncropped pictures of the Western blots shown in the manuscript in Fig 2F: Protein expression of TMEM150C (A) and ß-actin (B) in various tissues of *Tmem150c*$^{+/+}$ and *Tmem150c*$^{-/-}$ mice (see Fig 2G). Predicted molecular weight for TMEM150C is 27 kDa and for ß-actin 42 kDa. ß-actin expression was used as a loading control. 1: Epididymis, 2: lumbal DRGs, 3: Liver.
